# Book Reviews

**Published:** 1824-01

**Authors:** 


### CRITICAL ANALYSIS
OF
### ENGLISH AND FOREIGN LITERATURE
RELATIVE TO THE VARIOUS BRANCHES OF
### Medical Science.

Quæ laudanda forent, et quæ culpanda, vicissim

Illa, prius, cretâ; mox hæc, carbone, *notamus*.—PERSIUS.

### DIVISION I.

### ENGLISH.

ART. I.—*On the Nature and Treatment of the Distortions to which the Spine and Bones of the Chest are subject; with an Inquiry into the Merits of the several Modes of Practice which have hitherto been followed in the Treatment of Distortions.* By JOHN SHAW, Surgeon and Lecturer on Anatomy.—8vo. pp. 293. Longman, Hurst, Rees, Orme, Brown, and Green. London, 1823.

ART. II.—*Engravings, in folio, illustrative of a Work on the Nature and Treatment of the various Distortions to which the Spine and the Bones of the Chest are subject.* By JOHN SHAW, Surgeon and Lecturer on Anatomy.—London: Longman, Hurst, Rees, &c.

THERE is not a more gratifying circumstance in the history of medical science, than the character of observation it has of late years assumed. It is unnecessary to adduce proofs of this position: all must be convinced of it who are attentive to what is going on around them, and who extend their views beyond the sordid speculations of the trade, to the nobler interests of the profession. It is true that much is still wanted to rouse the spirit of research, and fix it on proper objects; but what has been already done gives us considerable hope that all received opinions, which are founded on names and supported by prejudice, will soon undergo a severe examination, from the inquisitive spirit of the rising generation of practitioners. Mr. CHARLES BELL has lately led the way in the road of discovery, and has opened the door to so much physiological and pathological research, that every thing which comes from his school has now a strong claim to the attention of the public. It was, therefore, with peculiar pleasure that we took up the present volume, from the pen of his brother-in-law, Mr. SHAW, in a new line of study; and our expectations have been fully satisfied by its perusal.

Mr. Shaw, in considering the diseases of the spine, has followed a plan somewhat new, but well adapted to the nature of the study, which has more to do with facts than speculations.

It is a plan which allows free scope for the vigorous method of investigation he pursues, and which brings him more quickly and more certainly to his conclusions. It is a plan which may displease those who love to *skim* books rather than to read them, and whose sickly appetite does not relish the wholesome food that is here set before them; but it will have double charms for those persons who rather choose to grapple with the rude facts of science, and to establish their opinions on a strong and immutable foundation.

Mr. Shaw tells us that he has, for some years, paid great attention to the deformities of the human skeleton: he has visited the different anatomical collections in London, Edinburgh, Glasgow, and the principal cities on the continent; he has compared the preparations preserved in these collections with each other, and with a very extensive museum in Windmill-street; —he has also endeavoured to collect the history of the persons from whom such preparations were taken, and from the which, along with extensive opportunities of observing the progress and effects of deformity in the living body, he has deduced the conclusions which form the ground-work of the volume we are about to consider. The book therefore teems with facts; and, when this is considered, our readers will, perhaps, see the propriety of the plan adopted by Mr. Shaw, of throwing his various and extensive materials into the form and order of demonstration, and seeking every assistance which diagrams and figures offer in aid of description. This is the more necessary, as he is led, by the nature of his subject, to deal much with the forms, connexions, and relative situations of bones, which, however familiar they may be to the teacher of anatomy and the diligent student, cannot be supposed to be ever present to the memory of the medical practitioner, in a manner equally distinct. The latter, however, will find great assistance from the diagrams, which make the science of detail quickly familiar to him. Some of the linear engravings, indeed, exhibit the deformity, with its cause, in a very happy manner.

There is one disadvantage, which most, if not all, the authors who have treated of this subject labour under. The opportunities of observing the appearances after death, in cases, of which they have watched the symptoms during life, have been extremely rare. It is, indeed, a disease seldom fatal by itself, and, when the decrepid die of other complaints, it is generally in the hands of physicians who are little anxious about the important subject which is here treated of, even though it may have contributed to exasperate and complicate the fatal disease. Here, however, the author has enjoyed a great advantage; his particular pursuits as an anatomist having been the means of throwing open to him a source of instruction denied to others; and we are glad to find that he has made use of it. It must be evident, indeed, that it is only by comparing the appearances after death with the history of the disease, of its origin, progress, and, more especially, of the means adopted for its relief and cure, that we can hope to arrive at the most enlightened views of its nature, and a knowledge of the proper course to be pursued for its alleviation.

In the pursuit of the subject of lateral curvature of the spine, which occupies the whole of this volume, our author has been led to treat of an important law in the animal economy. It appears that it holds equally true with regard to our physical powers and the structure of our bodies, as in the constitution of our minds and the disposition of our faculties,—that they are lent to us only for use, and, when unemployed, they quickly fall into decay. But that which appears very singular, is the fact that the substance of muscle, tendon, artery, and bone, are, when long disused, resolvable and resolved into the common cellular membrane. The facts in proof of this position, which have occurred to Mr. Shaw, are so numerous and so striking, that he cannot refuse his assent to the conclusion, and his reader will find great difficulty in explaining them on any other supposition.

"On first examining the parts (says our author,) which compose a joint, and each of which is so admirably fitted for its peculiar office, we do not at once acknowledge the truth of what has been stated. On the contrary, it is difficult to imagine that the bone, so curiously adapted for security and motion,—the cartilage, so different in appearance from the bone,—and the secreting membrane, so peculiar in its character,—should, with the dense ligaments, the tendons, and bursæ, be all resolvable into a matter similar to the cellular substance by which they are united to each other. But, however improbable this may appear, its correctness is easily demonstrated. As long as a joint is kept in activity, the apparatus continues perfect; but, when the motion of a joint has ceased for some time, all its complex parts degenerate; their peculiar character and structure disappear; they fall into the same condition, and assume the same appearance, with the cellular membrane. When we examine a joint which has been anchylosed, we see that the character of every part is changed. The bone is no longer hard, but softened and cellular; and the bursæ, the capsules, and the ligaments, form one indistinct mass of cellular membrane." (p. 3.)

The converse of this statement is found by Mr. Shaw to be equally true—

"That new organs, in appearance and in function, may be formed of cellular membrane. If a bone be dislocated, and its head lie imbedded in the cellular membrane, cartilages, capsules, bursæ, sheaths, ligaments, all may be formed from it; and if these parts, constituting a new joint, be kept in activity, although they may not have the regularity of the apparatus of the original joint, they assume all the characters of the several parts. Even the new cartilage is similar to the original matter, which is supposed to be a substance *sui generis*. The new capsule has also all the characters of the proper synovial membranes. Indeed, when we carry the inquiry further, we discover that all the membranes, which have hitherto been classed as distinct from each other, and supposed to be peculiar in structure, are not only resolvable into common cellular membrane, but may be formed from it." (p. 3.)

Such are the opinions of our author, which we have given in his own words. Many and various proofs and illustrations of them are contained in this work, from which it appears that the cellular membrane is the pabulum, the primordial matter, the common matrix, of all the organs of the body, and not "merely a modification of the original tissue, in which the peculiar substances that give character to bone, muscle, and nerve, are deposited, but even that from which other parts may be formed." (p. 2.) This subject seems to have occupied a considerable share of our author's attention, and some hints which are given regarding the changes which inaction induces in the different textures of the body, will be read with interest. But it has an immediate and important connexion with the various curvatures and distortions of the spinal column.

Mr. Shaw, in pursuing his investigations, has been led to conclusions exactly the opposite of those which have been adopted by most practitioners,—we mean with regard to the principle of endeavouring to counteract deformity by force, variously modified and differently applied. He has found that, by contending with a distortion in this manner,—by pushing in the direction contrary to the inclination of the vertebræ, the disease will almost invariably be increased.

Let it be remembered that, when patients have recourse to medical assistance for the removal of this unseemly disorder, that the bones have often become settled in their distorted situations, and that the only position in which the patient is comfortable is that ungainly one which is constituted by the deformity. The muscles, ligaments, and cartilages, have become relaxed on one side,—shortened, compressed, and corrugated, on the other. Every effort that is made to restore the spinal column to its proper erect station will, therefore, be attended with pain and difficulty. It is well known that all the collars and apparatus which are made by ordinary bandage-makers, are subject to this inconvenience. The muscles are naturally roused to struggle against machinery so irksome; but let any surgeon for a moment reflect on what set of muscles are thus called into action. Are they not the very muscles whose contraction tends to bind down and confine the spine to its distorted position? Such is the opinion of Mr. Shaw,—such must be the opinion of every one who allows himself to reflect for a moment; and, if such be the fact, it is evident that all the complex machinery, with which young women have been hitherto tormented, is incorrect in principle and injurious in practice.

But we anticipate. Before we enter on the consideration of the cure, we have first to go through the examination of the nature of lateral distortion of the spine,—to inquire what are the parts most interested in its production,—and what are its most common exciting causes.

Mr. Shaw first establishes the general principle, that whatever weakens the muscles of the back, by taking away one of the strongest supports of the spine, gives rise to distortion. In the enumeration of the various causes of this local debility, the first that strikes us is that of local paralysis, which on a former occasion was the subject of our author's researches. Mr. Shaw has found that this is not an uncommon disease in the muscles of the spine and of the inferior extremities, and that it has the effect of distorting the column from its natural erect position. It is frequently a disease of old date, and has not been discovered till the increase of the curvature occasioned a more accurate examination, and led to its detection. Of this Mr. Shaw gives the following instance:—"A boy, on coming home from school during the holidays, was observed to limp in walking, and to appear crooked while standing. On being stripped, the left shoulder seemed to be lower than the right; but, on further examination, this effect was seen to be produced by the oblique position of the pelvis. On making him stand up so as to bring the shoulders to the same level, the left heel was raised from the ground; and, on carefully examining the left leg by measurements, it was found to be much smaller in all its dimensions than the right." (p. 32.) Mr. Shaw is inclined to attribute the greater number of such cases of local paralysis to affections of the alimentary canal, having found many of them follow immediately after an attack of abdominal irritation.

The next cause of curvature of the spine mentioned by Mr. Shaw is habitual bad position in young people. This is so common a source of distortion, one so important, and so fully treated of in the work under consideration, that we recommend all those who are interested in the subject to peruse the original, of which our limits do not allow us to make a satisfactory abstract. We believe this to be the most common of all the causes of distortion, and that which is, in general, most unscientifically and cruelly treated. All classes of society unite in deprecating the absurd and injurious treatment to which young ladies are so frequently subjected while at school. Under the head of *bad position*, Mr. Shaw considers the effects of the various amusements of children; of the different occupations of girls, as drawing, writing, music, and dancing; of the habit of standing on one foot, so common among boys, of stooping, &c.; and under each of these heads he endeavours to trace the manner in which the spine becomes affected, and the particular changes induced. "If a weakly girl, of ten years old, be obliged to sit for hours on a narrow bench, without any support to her back, it is not surprising that, notwithstanding all the reproofs she may receive, she endeavours to relieve herself by allowing the lumbar vertebræ to sink to one side. This may of itself be sufficient cause for the origin of a curve; but, if the position in which girls generally sit, while writing, drawing, playing the piano-forte, and more especially the harp, be taken into account with the causes already mentioned, it will be admitted that it is scarcely possible for a girl so situated to avoid being crooked; particularly if she is not permitted to take such exercises as give tone and strength to the muscles of the spine." (p. 56.)

The last observation is the basis of all good practice in this complaint. Every person is aware how much more irksome and painful it is to sit or stand in the same posture for a great length of time, than it is to take even strong exercise. The most robust man feels very considerable fatigue in his back and loins, after having passed an evening at a concert or in the pit of a theatre, or after having stood a part of the morning in a picture-gallery. Yet we expect greater efforts than these from young women, whose bones are yet soft, whose muscles and ligaments are not yet strengthened by time, and indurated by exertion. They are placed on a high stool, and obliged, most unpitifully, to pass hours in a constrained position, their backs aching with exertion, and lamenting in their hearts the grievous necessity of becoming accomplished. It must be singularly unfortunate for them, indeed, if they do not find some opportunity of eluding the keen glances of their governess, and "of allowing the lumbar vertebræ to sink on one side." The fact is, that by far the greater number of them do so, and many thus become crooked.

It is a very common opinion, that curvature of the spine is most common in scrofulous habits. This opinion is contradicted in various parts of the work which forms the subject of this article: we regret, however, that it does not any where meet with a lengthened discussion. It is true that the account given so satisfactorily of the origin and progress of lateral distortion, is sufficient refutation of the opinion we have mentioned; yet the idea is so common, that we think a particular discussion of it might have been useful. It appears to us that this opinion has arisen from confounding lateral distortion with caries of the vertebræ; but, from whatever source it may have taken its rise, nothing appears more certain than its fallacy. The softened state in which the vertebræ are sometimes found in this disease, is considered a proof of the scrofulous diathesis being very highly marked. This is a gratuitous assumption, entirely destitute of proof. Mr. Shaw has most clearly shown that it is merely owing to the inaction in which the bones have been kept, and is never to be observed in those decrepid persons affected with lateral curvature, who have been able to use strong exertion, and have not been bed-ridden for a long time previous to their death.

Our author devotes a chapter to the refutation of the opinion that the undue and too-powerful contraction of the mass of muscular fibre, which clothes and protects the spine, is a cause of lateral distortion. It seems that this opinion, of German origin, has gained currency in London of late years. That it is incorrect, is sufficiently proved by the following passage, which does not seem to admit of any obvious reply:

"As the part of the spine between the origin and insertions of these muscles is almost invariably curved in two opposite directions, before we can agree in the opinion that lateral distortion depends on the irregular action of the muscles, we must believe that the lower portion of the muscle on the one side may be in forcible and unnatural action, while its upper part is relaxed; and that in the muscle of the other side the reverse takes place. But this cannot be admitted; and indeed, the more the theory is inquired into, the more erroneous it will be found to be. If the distortion depended on an undue action of the muscles of one side of the spine over those of the other, the curve would be always in the form of a single arch, instead of its being of a serpentine shape, as it generally is, between the points from which the muscles arise, and those into which they are inserted. It has been stated, by way of argument, in favour of the opinion, that the muscles on the concave side of the curve are stronger than those of the other; but I suspect that this statement is not founded on proofs derived from an examination of the muscles in the dead body, for I found those on the convex side to be the largest. Indeed, there is one circumstance in the condition of the nerves of a distorted spine, which would induce us to believe that the muscles on the concave side would be even more deficient in energy than those on the convex side; for the nerves which pass to them are diminished to less than one-half of their natural size."

The last of the causes of lateral distortion, taken notice of by our author, is disunion of the bodies of the vertebræ. This is a very common opinion, founded on the circumstance already noticed of the occasional softened state of the vertebræ. Mr. Shaw thinks it very necessary that this error should be removed, as it leads directly to a great mistake in practice. It is thus, he informs us, we see unfortunate patients condemned to pass weeks and months in the horizontal posture, adding to the disease it is intended to cure. The wasted and blanched condition of the muscles confirms the opinion that the true origin of this state of the spine is weakness and inaction, and that the only method of cure, which can prove effectual, is to pay proper attention to the general health, and to call into operation those parts which are suffering from want of use.

"In the reports which from time to time have been made on the condition of the vertebræ of persons who have died with lateral curvature of the spine, it is generally stated that the bodies are softened, and full of a scrofulous substance; but, on investigation, this will be found to be incorrect; for, in the greater number of instances, the internal structure of the bodies of the vertebræ has a natural appearance. It is easy to account for the mistake. If the vertebra of a patient who has long been confined to bed be examined, the appearance described above is found; but, if the person has been in the habit of taking exercise a short time previous to death, the bodies of the vertebræ are discovered to be as firm and compact as those in a perfect spine. I trust that it is now almost unnecessary to add, that, instead of admitting that the softened condition of the vertebræ affords a proof of the existence of a scrofulous disease, that it should be considered as merely showing the bad consequences of confinement and want of use. The wasted appearance of the muscles, which is occasionally observed in cases of lateral curvature, and which has been confounded with their state during the inflammatory stage of the carious diseases of the spine, also depends on the same causes as the softening of the bones.

"It is well known that the shape of the vertebræ is materially altered in cases where the spine is much distorted; but, as no mark of disease is discovered when a section of the bones so mis-shapen is made, we may infer that the change of form is a consequence that may be produced independent of any specific disease existing in the bones, especially as it is found to correspond to the direction in which the pressure has been made." (See page 93.)

The last subject discussed by Mr. Shaw, of which we intend to take notice, is one of very great importance. We allude to the condition of the pelvis in cases of lateral distortion of the spine. He has examined a very great number of cases of diseased spine, and he has uniformly found that, whenever the long bones were unaffected, no narrowing of the pelvis had taken place. Whether this holds universally, seems yet doubtful; but it is certain that it is true in by far the greater number of instances, and it would also seem to follow from analogy with the other facts contained in his book. If the disease of lateral distortion be separated from rickets, and shown to have no connexion with disease of the bone, we cannot see any good reason for suspecting the pelvis to be interested in the distortion. The fact, however, of there being not a single example of misshapen pelvis accompanying lateral distortion of the spine, either in the museum at Windmill-street, in any of the anatomical collections of the large hospitals, in any of the collection of public bodies or private individuals of this city,—in the museums of Edinburgh, Glasgow, Leyden, or Paris, seems quite decisive of this question, and to afford as extensive and conclusive a body of evidence as can be expected on any subject of medical science. If to this, indeed, be added the recorded cases, which all speak in favour of the same opinion, little doubt will remain that, where the only external evidence of distortion is lateral curvature of the spine, the pelvis is always of its natural capacity. Accoucheurs, indeed, have long suspected that narrowing of the pelvis was by no means a constant concomitant of, and indeed did not bear any proportion to the decrepid appearance of the external form. But it remained for Mr. Shaw to point out the great general principle which we have just explained, and to prove it by a well-connected chain of facts and reasoning.

The value of the above rule to midwives is incalculable, and we trust, when generally known, will be found most serviceable to humanity. Every person engaged in obstetrical practice is convinced that a great number of unhappy infants are annually sacrificed by rash and ignorant practitioners. Embryotomy seems to us an act from which the boldest must recoil, and which is calculated to make the most thoughtless reflect: yet it may sometimes be unavoidable,—and, with regard to many of our accoucheurs, we are convinced that, were they not supported by a sense of duty, the operation to which we have alluded would be equally afflicting to their sensibility as to their reason. How many evils follow a single false step in science! Dr. Hunter, whose experience on this subject will not be doubted, has been known to declare to his friends, that nothing gave him more uneasiness, or cost him more consideration, than his annual lecture on the instruments used in midwifery; so fearful was this celebrated physician of giving encouragement to their use: and, though he did not absolutely protest against their employment, it is well known to those who remember his admirable lectures, that he annually stated his belief that it were well for the happiness of mankind had they never been invented.

It has been long supposed that the pressure of stays and other instruments on the pelvis, is a frequent cause of its distortion. This opinion received the sanction of the late Mr. Wilson's name. It has been diligently examined by Mr. Shaw, and the result of his search is a conviction that it is ill-founded: he has not been able to find a single preparation which countenances it. The cases alluded to by Mr. Wilson are all of them ricketty affections, the preparations being still preserved in the museum in Windmill-street.

We now proceed to give some idea of the plan of cure pursued by Mr. Shaw, and we have already anticipated much of what we have to say on this subject. When the nature of a complaint, and the causes that have produced it, are well understood, the method of treatment becomes a very simple matter, in a great many instances. The great principle on which our author proposes to treat all cases of lateral distortion, is to call into habitual exertion those muscles which have a tendency to counteract the deformity. The exercise to which they are submitted must, of course, be proportioned to the strength of the subject, and must never proceed to such a degree as to become fatiguing: but it is wonderful how much exertion persons afflicted with the diseases we have been considering will bear after a little practice.

We find a very striking proof of the correctness of the opinion advanced by Mr. Shaw, in an extract from the travels of a German author, cited by Dr. BEDDOES, in his work on Consumption. This gentleman was struck with the strong and athletic forms of the skeletons still exposed on the field of battle at Murten, where Charles the Bold, with his Burgundians, fell a sacrifice to the patriotic valour of the Swiss. His expressions are these:—"The three hundred years during which they have been exposed, in great measure, to the open air, have little affected their prodigious firmness of structure. Such bones, and parts of bones, as now moulder down in a few years of exposure were evidently firmer than in the recent subject. From rubbing together in my box, they acquired here and there the polish of the enamel of the teeth. Out of the charnel-house at Murten, I selected skulls that attested the strength of the stroke by which, as appeared from the marks, the helmet was cleft, and which, being pierced in the orbits by the point of the spear, probably belonged to knights, since the spear would be directed against this as the most vulnerable part. I still possess these specimens, and I consider them as an incontrovertible answer to the question, how these knights could wear armour insupportable by the present race? They were more hardy and athletic than we are."—(*Ebell uber die Bleyglafur*, Hanover, 1793.)

This is a more rational account of the prowess of our ancestors than the incredible histories which would make us believe that we had degenerated from their outward appearance and gigantic stature. The truth is, that, as Mr. Shaw has stated, the bones become closer in their texture, the whole frame of the body is more intimately knitted, and greatly strengthened, by that life of exertion and fatigue which our forefathers led.

The common practice hitherto adopted for the cure of lateral curvature, has been the use of the inclined plane, and of various kinds of stays and collars. On the use and abuse of the former instrument, we extract the following passage from Mr. Shaw, which is replete with good sense, and is so important, that we feel confident it will be read with satisfaction. At the same time, we must say, that our own observation has not led us to form so unfavorable an opinion of the effects of the inclined plane upon the health, even when continued for several successive months.

"It is scarcely necessary to repeat the arguments that have been already offered in refutation of these views; [nor are proofs now required to show that every part of the body is weakened and deteriorated by lying dormant. But I may state, that it is scarcely possible to imagine any means so effectual in preventing parts from performing their natural functions, as the plan proposed for the cure of a disease originally proceeding from weakness. Indeed, this now begins to be discovered, and the use of the inclined plane is gradually falling into disrepute: for it is found that, although a girl, who is slightly distorted, may become more straight after having been confined to the horizontal position for months, she does not gain strength, but, on the contrary, becomes so weak, that she can scarcely walk or stand; and, when she attempts to sit without some artificial support, she sinks almost double, or at least in a state worse than she was in when she first lay down. These are sufficient objections to the practice; but the effects which a long continuance in the system has upon the general health, are still more serious. We find that girls, who have been long confined to the reclining board, are delicate, and liable to all the worst symptoms of hysteria; and I have already mentioned an instance of a young lady having so many symptoms of diseased heart, that she was treated accordingly, although they were afterwards proved to have proceeded from the weakness caused by long confinement to one position.

"But, in making these observations, I beg it may be distinctly understood that they have reference only to the common notion that close and strict confinement to one particular position is the most probable mode of remedying distortions. Indeed, in the chapters descriptive of what I suppose to be the best means of treating distortions, it will be found that I consider the use of the inclined plane as essential to the cure of lateral curvature. Nay, I would even recommend that a delicate girl, although she may not be in the slightest degree distorted, should lie for some time every day upon the plane. When a girl is growing rapidly, and is at the same time delicate, the weight of the upper part of the body is obviously so much more than the lumbar vertebræ can support beyond a certain time, that common sense dictates the necessity of giving ease and rest to the spine; and this cannot be more effectually or easily done, than by lying down occasionally on the inclined plane. But this, like many other useful remedies, has been abused, from not paying sufficient attention to the great variety of affections to which the spine is liable." (p. 157.)

Regarding the use of stays and collars, we find the same opinion expressed in this work which has been maintained by well-informed surgeons in all ages. Whether Mr. Shaw, by dissecting the evil a little more minutely, and exposing the source of its baneful effects, will be more successful in destroying it, is a matter of some doubt. It is hard to root out established opinions, however absurd; but every person who gives himself the trouble of considering the following passage, will find sufficient materials for making up his mind on the subject:

"If a lateral curvature of the spine be observed at its commencement, it may be easily cured, or at least prevented from getting worse, unless it depends on some specific disease of the bones. But certain erroneous notions on the means of preventing distortion are now so prevalent, that, although the practice founded on them is not only inefficacious, but even injurious, it is often impossible to overcome the prejudice of parents until the distortion of the spine has proceeded to a considerable degree. Another source of difficulty consists in this, that when the spine is distorted only so far as to produce what a mother calls *a bad carriage*, the means commonly used to correct it are exactly such as tend to increase it.

"For example, if one shoulder projects rather more than the other, or if one side seems a little larger than the other, a pair of stiff stays and a collar, to brace the shoulders back, are immediately applied; and this plan is persevered in, for every person in the family is delighted to see how much the child's figure is improved. But, although the evil may be concealed for a while, its cause is increased by wearing stays or a collar; for the child can no longer take that sort of exercise which is necessary to keep the muscles of the spine in such a state of activity as to fit them for their several uses.

"The examples to prove the necessity of exercise to the proper development of the different parts of the body, afford conclusive arguments against this plan of cure, and also against the practice of encasing children from morning to night in stiff stays. It is not necessary to bring these examples forward a second time; but, as it may be well to show how much the system of using artificial supports has been deprecated by men whose experience, and whose characters as anatomists, entitle them to be considered as authorities on all questions relating to the human form, I refer to the works of Riolan, Haller, Winslow, Van Swieten, and Portal.

"Portal has entered at considerable length into the inquiry: among other facts, he states that the muscles of the back are larger, redder, and stronger, in women who have not worn stays, than in those who have used them. He says, indeed, that it is scarcely possible to demonstrate the muscles of the back in those who have worn stays, or any similar contrivances, to support the spine." (p. 190.)

Mr. Shaw trusts the cure principally to exercise, so contrived as to call into action those muscles which have a tendency to counteract the distortion. Experience has taught him that the only effectual way of treating those cases, is by calling into operation the natural powers of the diseased part, which, by disease and inactivity, have been suffered to languish and decay. It is thus that a blow is struck at the root of the evil, and, by imitating nature, we are taught to cure her aberrations.

A great variety of means have been devised by him to execute this plan: many of them are exceedingly ingenious, but, without the assistance of his diagrams, we should despair of giving a correct idea of them to our readers. We must therefore refer them to the work itself, where they will find details sufficient to enable them both to understand and to apply them. The plates in illustration, reflect high credit, both on the judgment of the author, and the skill of the artist.

---

ART. III.—*Medico-Chirurgical Transactions, published by the Medical and Chirurgical Society of London.* Vol. XII. part 2.—London, 1823.

IN our account of this volume, we have separated the papers containing essays or general remarks from those consisting merely of individual cases: the former are placed in the same order in which they occur in the book, and the latter follow similarly arranged.

1. *On the Influence of local Irritation in the production of Diseases resembling Cancer, and other morbid Alterations of Structure.* By HENRY EARLE, Esq. F.R.S. &c. &c.

2. *On Chimney-Sweep's Cancer.* By H. EARLE, Esq. &c. &c.

It frequently becomes a question of the most serious importance, involving alike the reputation of the surgeon and the comfort, or even the life, of his patient, to decide on the expediency or inexpediency of an operation: and, although this remark applies with equal force to cases of extensive injury, from fractures and similar causes, yet, in the paper before us, the question is more particularly discussed in relation to cases of suspicious ulceration and hardness, similating malignant affections. In those fungoid degenerations of structure to which the notice of the profession was particularly directed by the late Mr. HEY, of Leeds, the almost invariable recurrence of the disease has confirmed the majority of surgeons in declining the hazard of any operation; and the same remarks apply with equal force to the true carcinoma, where the system has become unequivocally tainted.

It is not, therefore, in such cases that any degree of hesitation is likely to distress the mind of the surgeon. "Not unfrequently, however, (says Mr. Earle,) we meet with instances of disease which, although at first sight, they may put on so malignant an aspect as at once to deter us from proposing any operation, yet, on closer inquiry into the history of such cases, we shall often find that the morbid action has been first excited by some *local* irritation, and subsequently maintained by *local* circumstances." In some such examples it would appear that the constitution may be predisposed to disease, while in others it is only influenced by the continual irritation from a morbid condition of some particular part. That the extirpation of a part supposed to be affected with cancer, may be occasionally followed by complete and permanent success, seems as obvious as that true cancer may sometimes be confounded with a milder disease; and, although Mr. Earle may probably be right in his opinion that there are circumstances connected with their discrimination which are not sufficiently attended to, yet we confess that it is upon the correctness and applicability of our diagnostic characters that all the value of such discussions must ultimately depend. It was not, therefore, without some feeling of regret that we came to the conclusion of Mr. Earle's paper, without any such assistance as we had anticipated being afforded in the decision of the great question,—namely, the marks by which we may recognise a disease likely to be cured by extirpation, and one which is not: and we regret this the more, as the experience and professional acumen of the author would have led us to place much confidence in his observations.

*Diseases of the lips* occupy the first place in the paper before us, and probably afford the most frequent example of a disease purely local, exasperated by continual motion and other irritating causes, until it has assumed the appearance of cancerous affection. The following is the most striking example which has occurred to Mr. Earle.

"Mr. H. Webb, a native of the West Indies, aged sixty-four, called on me in June, 1816, to consult me respecting a disease at the angle of his mouth, which had commenced in December, 1814, with an induration which soon ulcerated, and had continued to make progress, unchecked by any application. During the whole of this period he had resided in the Bahama Isles. At the time of his calling upon me, the right angle of the mouth had ulcerated so far as to allow the lip to fall and to admit of a constant flow of saliva. The ulcer had a very irritable appearance, and occasionally bled; and around this, to a considerable extent, the cheek had a very firm scirrhous feel, and the glands under the jaw were enlarged. He suffered constant severe pain, and was scarcely able to introduce any food into his mouth, from the swollen state of the lips, and the pain it caused him. The discharge had a most fetid smell, and affected his breath so much as to make it very unpleasant to be near him. On minutely examining the part, it appeared to me possible to remove the whole of the disease, and by taking away the teeth in the lower jaw, which were rendered useless by the loss of all the corresponding ones in the upper, that the cheek and remaining portion of the lips might be brought into contact. I represented to him that I could not answer for the success of this operation,—that every thing would depend on being able to obtain union by the first intention,—and that, if we failed in this, it was more than probable the wound would never heal; I further stated that, if we succeeded in closing the wound, the relief might only be temporary. Still, however, I entertained hopes of a more prosperous termination. I recommended him not to abide by my decision, but to take the opinion of other surgeons. He applied to two of the ablest and most experienced in town, who both declined attempting any operation, in consequence of his age and the progress of the complaint, which they both considered as confirmed cancer. He returned to me, and told me what had passed: adding, at the same time, that he was willing to submit to any trial, however slight the chance of success; that I offered him a possibility of recovery, and the other gentlemen left him no alternative but to linger on a little longer, a disgusting object to others, and loathsome to himself. I requested him to take further opinions, but this he declined. On sending him to an eminent dentist, to remove the teeth in the lower jaw, that gentleman was unwilling to touch them, conceiving that the gums, which were very spongy, had taken the same diseased action. I mention these circumstances with a view to prove the malignant character of this disease."

The whole of the diseased portion, including the angle of the mouth and part of both lips, was removed; and the patient did well. "On examining the part after removal, it had the firm white bands and other characters of carcinoma."

In four other cases of diseases of the lower lip, less extensive but similar in kind, the author has removed the parts with success; and he states, as his opinion, that few cases afford better chance of fortunate issue than corroding ulcers, with scirrhous edges, occurring about the mouth. In such operations, as well as in that for hare-lip, Mr. Earle advises that the pin nearest the edge of the lip be first introduced, by which means the coaptation of the red margin of the lip is rendered more certain.

*The integuments of the nose and face* form the next section. The habitual use of snuff has been known by our author to produce a suspicious ulceration of the upper lip, which nevertheless has yielded to mild applications, and discontinuing the practice of snuffing. The irritation of shaving has likewise occasionally brought on a state of enlargement resembling cancer, but which, only *resembling* that disease, may frequently be removed by proper applications, or, at the worst, be extirpated with safety.

*Diseases of the tongue* are apt to be kept up by the frequent motion of the part, by which it is brought into contact with the teeth; and, if these be irregular or decayed, the chance of healthy action is diminished. In unfavourable cases of this nature, frequent recourse has been had to various operations with the scalpel, ligature, or cautery: the danger and suffering under such circumstances render these last resources of the art hazardous, and, if possible, to be avoided. Much here may be done, in the opinion of our author, by local and constitutional treatment.

"Under the head of local treatment, I would place in the first rank the removal, as much as possible, of all local stimuli,—such as the taking away any decayed or projecting tooth, the shielding the tongue from pressure by covering the teeth with wax or soft lint, the complete privation of the faculty of speech, the frequent cleansing of the mouth with a stream of water, or any medicated liquor, from an elastic-gum bottle, instead of gargling or washing the mouth by any muscular effort; the employment of the most mild unirritating food, and, in bad cases, the prohibition from the use of solid food, substituting in its room milk and strong broths, which may be thrown into the stomach through a tube passed down the œsophagus, and even, in some cases, introduced through the nostril. Such a plan of treatment requires much firmness and resolution on the part of the patient: but I can confidently state, that it will be occasionally followed with success. When there is any enlargement of the glands, it will be right to combine with this the repeated application of leeches under the chin. As local applications to the ulcerated surface, few are more efficacious than a solution of nitrate of silver, or very much diluted nitric acid, in the proportion of three or four drops to the ounce. Occasionally a solution of arsenic is very useful. Any of these may be thrown upon the ulcer with a syringe, which will be found better practice than keeping lint moistened with any lotion, constantly applied to the part."

Much benefit has appeared, to Mr. Earle, to arise from the extract of hyoscyamus, pushed to a considerable extent, (one drachm daily,) although other remedies of the same kind have been entirely unavailing.

*The affections of the prepuce* come next under consideration; and Mr. Earle is of opinion that, even when the removal of the diseased part becomes necessary, this may be successfully done, in the majority of cases, without having recourse to amputation of the penis itself. The powerful contraction which takes place during cicatrization renders it a matter of importance, in the after-treatment, to keep open the urethra by sponge tent; a plan which must be continued for several months.

Mr. Earle next touches upon the formation of *morbid growths,* particularly about the vagina and verge of the anus. The soothing plan of treatment is insisted upon throughout.

Mr. Earle's second paper relates to that peculiar species of cancer which affects chimney-sweeps, and which is thus described:

"The disease in question is invariably produced by the irritation of the soot applied to the rugæ of the skin. The characters of the complaint are peculiar, commencing in a warty excrescence, which in some cases will remain nearly stationary for several months, or even years. After a time, the excrescence discharges a thin acrimonious ichor, which excoriates the surrounding skin. Ulceration now takes place in the centre, and the edges of the wound become everted, and throw out a luxuriant growth, with schirrous hardness, which discharge a very fetid irritating matter. The most common situation for this complaint is the lower part of the scrotum; but this rule is not without exceptions: a remarkable instance of its occurrence at the wrist of a gardener, who was every spring employed to distribute soot for the destruction of slugs, is related by my late father, in the last edition of Pott's works. Some instances have also occurred where the disease has taken place in the integuments of the face. As the disease advances, the parts immediately contiguous become affected; it spreads downwards to the perineum, and, if not arrested, will include the whole scrotum; at the same time, it not unfrequently extends itself to one or both testicles. When the testicle is first affected, it becomes firmly connected with the diseased scrotum; it enlarges, and becomes greatly indurated; ulceration, and sometimes sloughing, then take place, leaving a deep excavated ulcer, that penetrates into the body of the testis, which does not appear disposed to the formation of fungous growth, similar to what occurs when the scrotum is the seat of disease. The same observation applies when the complaint has extended itself to the inguinal glands: its progress in glandular structures appears to be more rapidly destructive, without the slightest effort at reparation. The disease, in every instance that I have seen, except one, extended itself to the parts immediately contiguous. The inguinal glands are often enlarged, but they will generally subside on the removal of the diseased scrotum; clearly proving that the disease is not commonly communicated in the course of the absorbents. This is a very important feature in the complaint, and one which most materially influences the prognosis and treatment. I know but one exception to this rule, where a bubo formed, which suppurated, and the sore assumed the same characters as the primary affection in the scrotum. Even where the testicle is affected, the disease is confined for a time to that gland, but it subsequently extends itself up the spermatic chord, into the cavity of the abdomen, where it proves rapidly destructive. Not only is the discharge from the sore very fetid, but the perspiration from the whole body, which is generally copious, has a very peculiar ammoniacal odour, which cannot be mistaken when once recognised. The countenance has a peculiar leaden hue, and the general health is materially affected by the severity of the pain, the want of rest, and the constitution having to contend with a disease which it is incapable of throwing off."

Several cases are given in illustration.

2. *Cursory Remarks on Small-Pox as it occurs subsequent to Vaccination.* By George Gregory, m.d. Physician to the Hospital for Small-Pox and Vaccination at St. Pancras.

This subject is full of interest, but we have, in recent Numbers of this Journal, entered so fully upon the question, that we purpose at present to confine ourselves merely to those points of Dr. Gregory's paper which present features of novelty. It appears, from the statement before us, that the occurrence of small-pox after vaccination is greatly on the increase: in 1810, the proportion of cases of small-pox after cow-pox was, to the total number of admissions, as one in thirty; in 1815, as one in seventeen; in 1819, as one in six; in 1821, as one in four; and in 1822, as one in three and a half. This, indeed, presents an alarming picture; and we agree with the author, that it is only by a fair and open examination that the merits of the question can be determined, and the remedy (if there be any) applied. From this account it will be apparent that Dr. Gregory has enjoyed excellent opportunities of watching the phenomena of this disease under all its modifications, while the paper before us shows that these opportunities have not been allowed to pass without due advantage being taken of them. We beg to state further, that the paper is written in a cool and dispassionate manner, apparently without any pre-conceived opinion to be maintained,—a circumstance which, of course, entitles it to greater weight. It is now ascertained, beyond the possibility of reasonable doubt, that vaccination frequently fails to give perfect security against small-pox; nevertheless, this disease is, for the most part, modified by the previous vaccination. With regard to these modifications, the author shall speak for himself.

"1. Vaccination does not appear to lessen the violence, or shorten the duration, of the first or eruptive stage of fever, which is generally as severe, and even sometimes severer and longer in its duration, than that of the casual confluent small-pox.

"2. It does not appear, in like manner, to influence the *quantity* of eruption upon the skin, so much, at least, as has been generally imagined. It is true that, in many cases of small-pox, subsequent to vaccination, the eruption has been very scanty; but in a large number, also, I have seen it very copious, more particularly about the face, breast, and upper extremities; and occasionally fully equal, in point of *quantity*, to what is seen in the worst kinds of confluent or coherent natural small-pox.

"3. The great power of vaccination unquestionably consists in modifying the *progress of inflammation* in the variolous eruption; and here it cannot fail to attract observation, how strikingly opposed to each other, in this respect, are the influences of inoculation and vaccination. Inoculation lessens the *quantity* of eruption, but does not alter, in the slightest degree, the progress of inflammation in that which *is* brought out. Vaccination, on the other hand, while it does not sensibly affect the quantity of eruption, always influences, more or less, the progress of inflammation, however copious the eruption may be. The same desirable result, the diminution of mortality, is obtained in either way. By checking the quantity of eruption, or the degree to which inflammation in it extends, the disease is prevented from bringing on those impediments to the functions of respiration and perspiration which occasion secondary fever, and endanger life.

"In all, or nearly all, cases of natural and inoculated small-pox, the eruption proceeds to ulceration, more or less superficial, according to the violence of the disease; and the ulcers heal by the common process of scabbing and cicatrization. In cases of small-pox, however, subsequent to vaccination, the cutaneous inflammation is checked at so early a period, that the fluid in the vesicles seldom reaches the state of pus, the cutis vera is never ulcerated, and consequently the healing process takes place by the conversion of the vesicles into tubercles, and their subsequent desquamation. This constitutes a very well marked and important character of the vaccine or modified small-pox. A similar modification of the variolous inflammation of the fauces and trachea undoubtedly takes place; but the exact nature of the difference it is, in this case, more difficult to define.

"4. Though vaccination modifies, in a large proportion of cases, the progress of inflammation in the skin and throat, it is curious to observe that it does not always affect the course of the disease, when the variolous poison fixes itself on other parts, more particularly on the brain. It is in this manner that small-pox, after vaccination, occasionally proves fatal."

In the *degree* of modification, every imaginable gradation may be found in different cases, and we have elsewhere endeavoured to show the impossibility of distinguishing between mild cases of small-pox and severe cases of chicken-pox; there being in nature no distinct limit. It is satisfactory to observe how much the cases occurring at the hospital were mitigated by the previous vaccination; forty-four out of fifty-seven patients admitted under these circumstances, in 1822, having been discharged in perfect health *within fourteen days* after their admission.

The causes giving rise to small-pox after cow-pox resolve themselves into several heads.

1st. It appears, from the testimony of Dr. Gregory, and our own experience leads us to coincide with his observations, that a peculiar susceptibility to the disease exists in particular families.*

2d. It is stated, as worthy of observation, that the great majority of cases of this nature, which have occurred at the Small-Pox Hospital, have been in persons between fifteen and twenty-one years of age. From this circumstance the author is inclined to "entertain a notion that there is something in the habit of body peculiar to that age, which renders the system more than usually disposed to suffer from the influence of the variolous poison." We know not exactly what to say respecting this "notion" of the Doctor's; but we are inclined to think that the circumstance may be accounted for otherwise than by having recourse either to any peculiar small-pox propensities, at the time of life he has mentioned, or to the old doctrine that the influence of the cow-pock becomes exhausted after a certain indefinite and indefinable period. In the first place, children are very seldom taken to an hospital, either for small-pox or any thing else; and we can assure him that, if he directed his attention to some of the public institutions in this metropolis, which are peculiarly set apart for young patients, he would find reason to believe that small-pox after vaccination was not most pre-

* One of the worst cases of small-pox after vaccination we ever witnessed occurred in a boy whose father had had small-pox twice.

valent at the period of life he has supposed. It further appears, from Dr. Gregory's own account, that a great majority of his patients were persons who had come from the country; and when we consider that the class of persons who resort in sickness to our hospitals, are such as do not come to London till they are able to support themselves by labour, service, and similar occupations, we see at once why so few were under fifteen. Why the disease did not occur in persons more advanced in life is obvious,—viz. that they could not have enjoyed the benefit of vaccination; and, consequently, it is only from twenty, or at most twenty-five, downwards, that we can look for such cases.

The third point is one of great importance,—it relates to the connexion between the vaccine cicatrix and the variolous eruption: where the former is small, distinct, circular, and radiated, the latter is so slight as scarcely to merit the name of a disease. But, when the cicatrix is large, and appears to have been influenced by local inflammation, the secondary affection is much more likely to prove severe. This observation, which we have not elsewhere seen so expressly laid down, appears to us to be correct. Within this month we have attended some young ladies, in a boarding-school, affected with small-pox after vaccination, and the only severe case occurred in a girl who had a large unsatisfactory scar. We had ventured, from the commencement, to predict that she would have it more severely than the others.

4th. The large proportion of cases which have occurred in persons vaccinated in the country, has led Dr. Gregory to believe that this process is frequently very imperfectly conducted: he is further confirmed in his opinion, "first, from having been able to trace several cases of small-pox after vaccination to particular villages, in counties bordering on the metropolis; and, secondly, from having observed that a great proportion of those admitted into the Small-Pox Hospital, after country vaccination, had *large*, *irregular*, and therefore *imperfect* cicatrices."

Some further particulars will appear from the following tables:

"No. II.—*Table of the ages of the different patients affected with small-pox after vaccination, who have been admitted into the Small-Pox Hospital during the last five years.*

| | | | | | |
|---|---|---|---|---|---|
| Under 10 years of age, | | 5 persons. | At 20 years of age, | | 18 persons. |
| At 11 | — | 2 | 21 | — | 13 |
| 12 | — | 1 | 22 | — | 9 |
| 13 | — | 2 | 23 | — | 10 |
| 14 | — | 5 | 24 | — | 9 |
| 15 | — | 3 | 25 | — | 4 |
| 16 | — | 7 | 26 | — | 3 |
| 17 | — | 14 | 27 and upwards, | | 7 |
| 18 | — | 13 | | | |
| 19 | — | 11 | | | 136 |

"No. III.—*List of patients having small-pox after vaccination; distinguishing those vaccinated in the country from those vaccinated in town, and those not ascertained.*

| Year. | *Vaccinated.* In the country. | In London. | Not ascertained where. | *Total vaccinated.* |
|---|---|---|---|---|
| 1809 | 3 | 1 | 0 | 4 |
| 1810 | 4 | 0 | 1 | 5 |
| 1811 | 5 | 0 | 1 | 6 |
| 1814 | 4 | 0 | 0 | 4 |
| 1815 | 5 | 1 | 0 | 6 |
| 1818 | 7 | 0 | 2 | 9 |
| 1819 | 11 | 1 | 5 | 17 |
| To Sept. 7. | | | | |
| 1820 | 6 | 1 | 18 | 25 |
| 1821 | 19 | 2 | 7 | 28 |
| 1822 | 35 | 6 | 16 | 57 |
| Total | 99 | 12 | 50* | 161 |

"No. IV.—*Table, exhibiting the duration in hospital of the cases of small-pox after vaccination, admitted into the Small-Pox Hospital during the year* 1822.

| | | |
|---|---|---|
| Less than 7 days, | . . . . . | 12 persons, |
| — 14 — | . . . . . | 32 — |
| — 21 — | . . . . . | 6 — |
| — 6 weeks, | . . . | 2 — |
| Fatal cases | . . . . . . . . | 5 — |
| Total | . . . | 57" |

3. *On the Comparative Virtues of different kinds of Sarsaparilla.* By Mr. John Pope.

There are three kinds of this drug to be found in the London market,—viz. the Lisbon, Honduras, and Vera Cruz. Of these the Lisbon has been esteemed the best. Its characters are, externally, "a reddish or dark-brown coat; when cut longitudinally, it has a white farinaceous appearance, and is usually more free from chumps and fibre than the other kinds." The Honduras is of a dirty brown, sometimes having a whitish coat; "it is not so red as the Lisbon, is usually more fibrous, and possesses more pith." The Vera Cruz is inferior to the other

* "There is every probability that the fifty persons set down in this column were vaccinated in the country; as residents in town would hardly have expressed any doubts on the subject."

two, being dark and fibrous. Another kind of sarsaparilla has of late years been brought from Jamaica. "It has a peculiar, deep-red external coat, is of somewhat close texture, and, when cut longitudinally, that part of the outer coat which we designate as pith in the other kinds, is found more or less of a deep-red colour."

The medical efficacy of sarsaparilla resides in the bark, and consists of extractive; nearly the whole of its virtues are given out by cold infusion in distilled water, or in lime water, and are entirely extracted by the former when at the boiling point. The red variety, lately brought from Jamaica, yields by far the largest quantity of extractive.

4. ***On the Occurrence, in Persia, of the epidemic Cholera of India.*** By JOHN CORMICK, Esq.

The description of this formidable disease, given in the paper before us, differs little from the numerous accounts already before the public. Calomel and opium, separately and conjointly, are recommended in the commencement, and purgatives in a more advanced stage. "In many cases recovery was so slow, that strong purgatives were necessary every five or six hours, for two or three days." The author found more benefit from pieces of blanket moistened with hot water, and tied about the arms and legs, than from any other external application.

5. ***Some Observations on the Powers of the Circulation, and the State of the Vessels, in an inflamed Part.*** By A. P. W. PHILIP, M.D. F.R.S. ED. &c.

This paper contains so many references to the former writings of the author, that we, in imitation, must beg to refer to our former analyses of these. The subject is one not admitting of an account so condensed as our restricted limits would oblige us to make it. We suspect, too, that most of our readers are tired of hearing how the blood circulates in the web of the frog.

Tried experiments on frogs & dogs, & hogs

[To be continued.]

& magpies & [illegible]

### DIVISION II.

### FOREIGN.

ART. IV.—*De l'Influence du Système Nerveux sur la Digestion Stomache.* Par MM. BRESCHET, M.D.P. Chef des Travaux Anatomiques de la Faculté de Medicine de Paris, &c.; H. MILNE EDWARDS, D.M.P.; and VAVASSEUR, M.D.P.—(*Mémoire lu a la Societé Philomatiques,* le 2 Août, 1823.)

*On the Influence of the Nervous System upon Digestion, &c. &c.*

THE experiments and observations contained in the Memoir now before us, are so interesting in themselves, and of so recent a date, that we cannot, we conceive, perform a task more acceptable to our readers, than that of giving a succinct account of the question here discussed, and an exact translation of the experiments which MM. BRESCHET, EDWARDS, and VAVASSEUR have performed, for the purpose of clearing up many points hitherto the subject of much discussion and contest.

The section of the eighth pair of nerves is one of the most ancient physiological experiments with which we are acquainted, and the phenomena produced by their division evidently showed that the brain had great influence over the functions of the lungs and stomach, which are indeed principally supplied with branches of these nerves. Passing in silence over a long list of authors, whose inquiries were principally directed to the effects produced by the division of the eighth pair of nerves upon the lungs, the voice, and the action of the heart, and noticing only those who have paid attention to the result of their division on the stomach, we find Baglivi and Haller both remarking nausea and vomiting as a common consequence of this operation; and recent observers have fully confirmed the truth of their statements. Upon this point there is no difference of sentiment, but physiologists continue still to be much divided in opinion as to the effects which the division of these nerves produce upon the digestive power of the stomach. In making experiments relating principally to other physiological points, M. Ducrotay de Blainville, in the year 1808, and Mr. Brodie, in 1811, came to the conclusion that digestion was impeded, if not entirely prevented, by the division of these nerves; and Le Gallois appears to be decidedly of the same opinion, as far at least as concerned some of the smaller animals experimented upon. Dr. Wilson Philip, however, pursued this inquiry much further than those who had gone before him; for he not only announces the result of the section of these nerves to be a total extinction of the digestive powers, but that the destruction of the lower portion of the spinal marrow produces the same effect, and he also discovered a more important fact,—namely, that, in order to restore the powers of digestion, it was only requisite to cause a continued current of galvanism to pass by the inferior extremities of the divided nerves for the space of a few hours. These facts were deemed of such importance, that the Royal Society, to whom Dr. Philip's paper was presented, thought proper to appoint a commission, composed of three of its members, to repeat these experiments. The result of these inquiries was not favourable to the conclusions deduced by Dr. Philip: on the contrary, a kitten, in whom the nerves of the eighth pair had been carefully cut at the cardiac extremity of the stomach, did not appear to suffer from the operation: at the end of eight days it was killed, having three hours previously eaten some meat, —digestion was found greatly advanced, and the lacteal vessels filled with chyle.

M. Magendie and Mr. Broughton were both of opinion that, though the section of these nerves might render chymification more difficult, that the loss of the digestive power of the stomach was by no means necessarily the consequence.

Such was the state of this important question in the year 1821, when an account was read to the Royal Society, by Dr. Philip, of some experiments performed in the presence of Messrs. Brodie, Broughton, and others, in order to ascertain how the different results of their respective experiments could have arisen. The following conclusions may be deduced from this inquiry, and in the correctness of which conclusions all these gentlemen are now perfectly agreed.

1st. The simple section of the pneumo-gastric nerves is not sufficient entirely to destroy the action of these nerves on the stomach, and consequently will not entirely impede digestion.

2dly. This function is however interrupted, if, after having divided the nerves, their extremities are so disposed as to prevent their contact, or change their direction.

3dly. A continued galvanic stream, transmitted by the inferior portion of the nerve, appears to restore the nervous influence; for, in this case, there are no efforts made to vomit, and the food undergoes, at least to all appearance, changes similar to those produced under common circumstances.

These results prove that the difference of opinion as to the influence of these nerves over the faculty of digestion, arose from the manner in which the experiments had been performed, and upon the interruption, more or less complete, of the nervous influence.

The authors of the Memoir before us proceed to state that, without having the least doubt as to the accuracy of the above experiments, they considered it proper to repeat them, in order to assure themselves of the truth of the facts, and thus to obtain a sure foundation for their ulterior researches. They then detail their manner of conducting these experiments; the only points of which necessary for us to notice are these,—that, having first caused the animal to fast for twenty-four hours at least, they then gave it a certain quantity of food, and immediately proceeded to the operation, by dividing the integuments of the neck in the central line longitudinally, and turning aside the muscles until they reached the carotid artery, behind which the nerves to be divided are placed. By proceeding in this manner, the wound was regular, the bleeding very trifling, and the pain next to nothing. In the horse, it was necessary to make an incision on each side of the neck. In all their experiments with the pneumo-gastric nerves, that branch of the great sympathetic nerve which was closely united with those nerves was always divided. When the operation was finished, the animal was not allowed to take any nourishment; and, at the end of a certain number of hours, depending upon the age and species of animal, it was killed. To avoid circumlocution, they call the mere division of the nerves simple *section*, to distinguish it from that in which a portion of the nerve is removed.

The experiments detailed by our authors completely confirm Dr. Philip's statements, with this exception, that in the magpie they found, to their great astonishment, notwithstanding the division of these nerves, and an excision of part of their extremities, that digestion was completed, and that the respiration appeared to be but little affected. But an examination of the distribution of the par vagum in these animals showed that these nerves communicated with the neighbouring branches by means of numerous anastomoses, and they therefore regard this circumstance as accounting sufficiently for this anomaly.

Dr. Philip, having concluded from his experiments that the destruction of the lower part of the spinal marrow interrupted the process of digestion, was lead to suppose that whatever weakened the nervous influence would necessarily tend to diminish the digestive powers of the stomach. Our authors, directed by this hint, removed a certain portion of the hemispheres of the brain in a dog, and the most marked effects were produced upon its digestion. Eight hours after the operation, the food was less changed than in another animal, in which the simple section of the eighth pair of nerves had been performed; and sensibly more so than in another in which this section had been performed, and portions of the nerves removed. This effect was more marked in a small magpie, whose whole brain was removed, and the surface cauterised, in consequence of hemorrhage. Another method of diminishing the nervous energy was then tried, by placing the animal under the stupifying effects of opium. This was done by injecting the aqueous extract of opium into the veins of a dog, so as to produce a state of profound coma: at the end of eight hours, the food, which he had taken immediately before the experiment, retained its natural appearance in the centre; whereas, in another dog, upon whom no experiment had been performed, digestion was completed, and nothing remained in the stomach but a trifling quantity of chyme, of a greenish brown colour, and exhibiting merely some few fragments of food.

Dr. Philip has the merit of having first endeavoured to restore the action of the stomach, arrested by the section of the par vagum, by passing a galvanic stream through that viscus; and he concludes, from numerous experiments, that electricity is capable of replacing the nervous influence in the process of digestion. Messrs. Brodie and Broughton found that in a rabbit, submitted to the influence of galvanism for a certain number of hours, digestion did not appear to be more advanced than in a rabbit whose pneumo-gastric nerves had been divided; but our authors do not consider this experiment conclusive, because they have often found, in healthy and sound rabbits, that the food will remain entirely unchanged, even after it has remained for seven or eight hours in the stomach. In order to arrive at some decisive conclusion upon this point, they therefore performed the following experiments.

Having raised the pneumo-gastric nerve on the right side, for the space of two inches and a half or three inches, in a horse, which had just eaten a quantity of oats, the same nerve on the left side was divided, and the inferior extremity isolated for a certain length; a large opening was also made in the trachea, to prevent the occurrence of asphyxia, which in these cases frequently causes the animal to die almost immediately. The inferior extremity of the divided nerve was then surrounded with a thin plate of lead, which communicated with a trough containing fifty pairs of plates, of about six inches. The galvanic circle was completed by introducing into the abdomen of the animal, below the stomach, another plate of lead, armed with a conductor communicating with the opposite pole. During the whole of the experiment, slight contractions of the panniculus carnosus demonstrated the constant action of the galvanic current, which was kept up in this manner for seven hours. At the same time, the pneumo-gastric nerves of another horse, placed precisely under the same circumstances with regard to food, were divided; and a third horse, also fed in the same way, was left untouched, in order to enable them better to appreciate the influence of galvanism upon the digestion, by thus having two opposite points of comparison.

The results of these experiments were most marked. In the sound animal, digestion was completed; the small intestines were filled with a serosity of a clear yellow colour; the lacteals were gorged with chyle; and the cœcum was distended with a great quantity of oats, the greater part of which was greatly altered, the remainder not broken down. In the animal which had been exposed to the galvanic influence, the stomach, but little distended, contained a small quantity of liquid, and of oats slightly altered; all the rest had passed into the cœcum, where the grain was found, for the most part, very much softened, and reduced into chyme, mixed with some balls of oats, and with a liquid; but in a less quantity than in the sound animal. In these two instances the food, so altered, presented an odour very much resembling malt. The stomach of the horse in which the pneumo-gastric nerves had been cut, was very much distended; it contained a good deal of clear liquor, of a yellowish colour, in which was steeped nearly triple the quantity of oats that remained in the stomach of the animal that had been galvanised. The greater part of the grain had undergone no alteration; a small quantity only appeared half digested. They found in the cœcum nothing but an aqueous liquid, and some remains of straw, evidently the relics of a prior digestion. Thus it is evident that, in this animal, none of the oats, which he had eaten after fasting for twenty-four hours, had passed the pylorus; whilst the whole of the food in the sound animal, and at least two-thirds in the galvanised one, had passed into the intestines.

The experiment was repeated in the same manner upon two dogs, only employing batteries of less force. It lasted eight hours, and the results which we obtained were equally satisfactory. In one of these animals, the stomach contained a great quantity of meat, very much softened, the nature of which might, however, be ascertained by examining the centre of the mass, but which, both upon the surface and near the pylorus, was converted into a sort of homogeneous and semi-liquid pulp. In the other, digestion was still further advanced; the meat was reduced into a semi-fluid pulp; the centre mass had become homogeneous, except a piece of tendon, which was not changed.

In a third dog, whose eighth pair had been divided with loss of substance, they found in the stomach a mass of meat nearly dry, a little changed on the surface, and a little softened within, but preserving its fibrous appearance and red colour.

"Such (continues our authors,) are the facts which we have established relative to the influence of the nerves upon digestion. Our experiments, without being original, appear to us to offer some points of interest to physiologists; for the labours of those who have preceded us, in consequence of their great importance, required to be confirmed anew.

In comparing the results of the experiments noticed in this Memoir with each other, the following conclusions may be drawn:—

1st. The simple section of the pneumo-gastric nerves in the neck, without loss of substance, and without altering the position of their extremities, does not prevent digestion from being performed; it only retards it in a very evident manner.

2dly. The division of these nerves, with a loss of their substance, considerably diminishes the digestive action of the stomach, but does not appear entirely to abolish it.

3d. The division or destruction of the lower part of the spinal marrow, or the removal of a portion of the brain, is productive of the same effects as to the alterations which food undergoes in the stomach.

4th. Narcotics, administered in such a manner as to produce coma, equally diminish the digestive power.

5th. Every thing that diminishes the sum of the nervous influence transmitted to the stomach, impedes digestion.

And 6thly. When digestion is completely suspended by the section of these nerves with loss of substance, that action may be re-established by means of galvanism, and the food contained in the stomach converted into chyme, with almost as much rapidity, and to all appearance as completely, as under ordinary circumstances.

These conclusions differ but little from those which Dr. Philip has deduced from the series of experiments which he made with Mr. Brodie, &c. Nevertheless, although they render quite evident the influence of the nerves upon the phenomena of digestion, yet we do not accord entirely with the opinion of that physiologist, who regards that function as *entirely* depending on the nerves: in fact, we are lead to believe that the nervous influence is not the sole cause of digestion, although it is one of the most important agents in the performance of that function."

### *Summary of the Experiments.*

*Experiment* A.—On the 4th July, 1823, a gelding, height one metre fifty cent. and aged ten years, having fasted for more than twenty hours, ate about a quart of oats, and drank a little water. At a quarter-past twelve, the two pneumo-gastric nerves were cut, without isolating them from the neighbouring cellular tissue, and without any loss of substance. Immediately after the operation, respiration became very laborious; some minutes after, the animal appeared upon the point of suffocation; the trachea was largely opened, and immediately all the distress ceased. During the whole day, respiration was easy, the animal was tranquil, and appeared only a little depressed. At seven o'clock in the evening it was killed, by opening the great vessels of the heart.—*Examination of the body:* Stomach distended, and containing a good deal of yellowish liquid, a great quantity of oats half digested, pultaceous, and having a strong smell of malt; some grains appeared only softened, without being otherwise altered. Small intestines containing a little greenish-yellow mucus. Cœcum distended with an aqueous liquid, of a clear yellow colour, mixed with a certain quantity of the remains of straw. Lacteal vessels not at all distended, and containing little or no chyle. Lungs of a deep rose colour; the pulmonary cells distended, (the *pulmonary emphysema* of M. Laennec,) and containing a small quantity of fluid.

*Experiment* B.—The same day, a stallion, of the height of one metre fifty-five cent. aged twelve years, having fasted for twenty hours, ate, at the same time with the preceding animal, the same quantity of oats, and also drank a small quantity of water. At a little after seven o'clock, he was killed in the same manner, in order to be compared with the former.—The stomach did not appear distended; it contained greyish and mucous liquid, no bile, and no traces whatever of undigested food. The small intestines containing a great deal of chyme, of a clear yellow colour. Cœcum filled with a quantity of oats, very much altered, mixed with some balls containing grains not changed, and a little liquid. Lacteal vessels gorged with white and milky chyle.

*Experiment* C.—The 6th of July, at ten o'clock in the morning, the pneumo-gastric nerves of each side of a large-sized hound were cut, without loss of substance, and without isolating them from the cellular substance. The animal had fasted twenty-four hours, and had then eaten some boiled beef immediately preceding the experiment. He appeared feeble and depressed, continued lying down all day, from time to time making slight attempts to vomit, but without rejecting any portion of what he had eaten; his breathing but little affected. At seven in the evening he was hanged.—Stomach large, and containing a great quantity of meat, a portion of which, near the pylorus, was very much softened, almost pulpy, and homogeneous; the rest of the meat presented no kind of alteration: there was scarcely any fluid contained in the stomach. The alimentary mass was dry, excepting towards the pylorus, where it was reduced into a pulp sufficiently moist. Lacteal vessels apparently empty.

*Experiment* D.—The same day, at the same hour, a middling sized spaniel, placed under the same circumstances with regard to food as the preceding animal, had the pneumo-gastric nerve on each side divided, and about two inches of each nerve removed. The animal appeared upon the point of being suffocated; he is untied, and comes a little to himself. Respiration continues much impeded; repeated efforts to vomit continue the whole day, and occasionally a small quantity of greyish liquid is brought up. Hung at seven in the evening.—Stomach containing meat a little changed upon the surface, and preserving, within, its fibrous appearance and natural colour; the mass dry, and the stomach containing no liquid. Lacteals empty.

*Experiment* E.—A black dog, of a small size, had fasted for twenty-four hours; he had then some dressed meat given to him, and ten hours afterwards he was hanged.—Stomach containing only a little mucous liquid, of a greyish colour, but without any fragment of food unchanged. Lacteals filled with chyle.

*Experiment* F.—On the 25th May, a dog, of a small size, after having fasted for twenty-four hours, ate a certain quantity of boiled meat, at twenty minutes after eleven o'clock: immediately after it had eaten, the two pneumo-gastric nerves were divided, without loss of substance, and without isolating them from the cellular tissue. Efforts to vomit,—respiration but little troubled; nevertheless, the animal appeared to be suffering a good deal. He was killed at six in the evening.—Stomach filled with meat, but little altered near the cardiac orifice, in a half-pultaceous state near the pylorus. Lacteals containing a little chyle. Lungs sound.

*Experiment* G.—On the same day, the section of the pneumo-gastric nerve was performed on another dog, under the same circumstances with regard to food, and the two extremities were turned back upon themselves, by means of ligatures slightly tied. This experiment was performed at the same hour as the preceding one. Violent and repeated efforts to vomit ensued, but the respiration was nearly natural. At six in the evening the animal appeared to be suffering greatly, and it was killed.—Stomach full of meat very little changed, even near the pylorus. Lacteal vessels containing a little chyle. Lungs sound.

*Experiment* H.—On the same day, and at the same hour, the section of the par vagum was performed on a small dog, and an inch of each nerve was removed. The animal had fasted as long as the others, and ate at the same time with them. Violent efforts to vomit. At six in the evening he appeared to suffer much, and was killed.—Stomach containing some meat, softened towards the pylorus, and not at all altered towards the cardia. Lacteal vessels but little observable. Lungs sound.

An animal of the same size as the preceding, placed under the same circumstances, in order to serve as a point of comparison, was killed at the same hour.—Stomach containing some meat, almost entirely reduced to a pulp towards the pylorus, very much softened near the cardia, but preserving still a fibrous appearance. Lacteals filled with chyle. Lungs presenting some blackish spots, but otherwise healthy.

*Experiment* I.—On the 8th June, a small dog, having fasted for twenty-four hours, ate some boiled meat half an hour before the experiment. At twelve o'clock, both the pneumo-gastric nerves were cut, and more than an inch of each removed. At half-past eight o'clock he was hanged.—Stomach distended with a great quantity of food: the meat was softened, especially at the surface, but the greatest part preserved its fibrous aspect and natural appearance. Lacteals not visible.

*Experiment* K.—On the same day, the section of the spinal marrow, at the lower part of the dorsal region, was performed upon a small dog, placed under the same circumstances as in the preceding experiments, at a quarter-past twelve o'clock. A complete paralysis of the lower extremities immediately ensued, both with regard to feeling and motion: in other respects the animal did not appear to suffer much. No remarkable symptom was observed. At half-past eight o'clock he was hanged.—Stomach containing a great deal of meat, a little softened near the pylorus, but not sensibly changed near the cardia. The centre of the mass had undergone no alteration. Lacteals empty. The spinal cord had been completely divided between the second and third lumbar vertebræ.

*Experiment* L.—A small dog was hung at the same time with the preceding, in order to be compared with them. He ate at the same time, and had fasted the same number of hours previously.—Stomach empty, containing a certain quantity of meat reduced into a pulp, almost homogeneous, and nearly digested. The volume of the food, compared to that found in the Experiment I. is nearly as 1 to 15. Lacteal vessels filled with chyle, of a very white colour.

*Experiment* M.—On the 18th July, the section of the spinal marrow was performed on a small dog, at the lower part of the dorsal region. The animal had fasted as usual, and eaten just before the operation, which was performed at ten in the morning. Immediately after the division, complete loss of feeling and motion, but no other remarkable symptoms. Hanged at half-past eight o'clock.—Stomach distended with a good deal of meat: towards the pylorus the mass is a little softened, but generally it had undergone no alteration. Lacteals but little apparent. The division of the cord had been complete.

*Experiment* N.—A dog of the same size, all other circumstances being the same, was hanged at the same hour. Stomach contained some fragments of meat changed to a pulp, and a greyish kind of chyme. Digestion, as is commonly the case, was furthest advanced near the pylorus. Lacteals filled with chyle.

*Experiment* O.—The same day, at half-past eight o'clock in the morning, the section of the pneumo-gastric nerves, with loss of substance, was performed on a dog of a very large size. The animal had fasted forty-eight hours, and at eight o'clock had eaten a great deal of tripe and boiled meat. He continued lying down all day, and appeared much depressed. Respiration was a little impaired; he did not exhibit any inclination to vomit. He was killed at half-past eight o'clock in the evening, by opening both the carotids.—Stomach distended by a great quantity of large morsels of meat and tripe; the mass did not appear changed, excepting towards the pylorus, where it was a little softened and mixed with bile: in other parts of the stomach it preserved its natural appearance, even at the surface. Lacteals empty. Lungs a little hepatised, in comparison with those in former experiments.

*Experiment* P.—On the 3d of June, a shepherd's dog having been suffered to fast for twenty-four hours, a certain quantity of boiled meat was given to him, at twenty minutes after twelve o'clock, and, immediately afterwards, four grains of the aqueous extract of opium, dissolved in two drachms of water, were injected into the crural vein. Five or six minutes afterwards, the animal fell into a profound state of coma, and appeared insensible: at four in the afternoon, these symptoms had entirely disappeared. The same quantity of opium was then injected into the crural vein of the opposite side. The coma showed itself again, and continued until the animal was hung, at eight o'clock in the evening.—Stomach filled with meat preserving its natural appearance, although a little softened in the centre, and reduced into a pulp at the surface. Lacteal vessels containing a little chyle.

*Experiment* Q.—The same day another dog, circumstanced with regard to food in the same manner, was hanged at the same time with the preceding.—Stomach almost empty, containing only a few small fragments of meat, and nearly two spoonsful of a greenish brown liquor. Lacteals filled with chyle.

*Experiment* R.—On the 20th July, the pneumo-gastric nerves of a young guinea-pig, which had fasted for twenty-one hours, were divided, with loss of substance, at ten in the morning, immediately before which he had eaten some cabbage-leaves. The animal appeared depressed and low; he died about eight hours after.—Stomach containing the cabbage-leaves much divided by mastication, but which did not appear to have undergone any alteration, preserving both their natural colour and odour. Lacteal vessels not visible.

*Experiment* S.—The inferior portion of the spinal marrow of another guinea-pig, placed under the same circumstances with regard to food, was disorganised by introducing a stilet into the spinal canal. In a moment the hinder limbs were paralysed, but soon after the operation the animal did not appear to suffer. At seven in the evening he was killed, and the stomach was found in the same state as in the preceding experiment.

*Experiment* T.—On the 19th June, a young pigeon (*columbia livia*, Brisson,) was crammed with vetches, having fasted for twenty-four hours. Immediately after, the simple section of the par vagum was performed at twelve o'clock. The animal appeared weak and depressed, opened its beak a little; the inspirations were slow and deep. Killed at half-past ten o'clock. —Crop filled with vetches, scarcely moist. Gizzard containing a few grains of vetches, softened, but seeming otherwise but little changed; others were deprived of their proper envelope, and separated into two; a small quantity of a substance, of a clear green colour, was also found.

*Experiment* U.—A pigeon of nearly the same age, not yet feeding alone, was placed under the same condition as to nourishment as the last. The section of the same nerves was performed at a quarter-past twelve o'clock, and about half an inch of each was removed. The animal opened its beak widely, and appeared to suffer much more than the other. It made several efforts to vomit during the day; at half-past ten o'clock it was killed.—Crop filled with vetches, unchanged, and more dry even than in the preceding case. Gizzard containing only some fragments of grain, and the green matter above mentioned.

*Experiment* V.—A third pigeon, of the same age, was chosen for the sake of comparison. It was treated, with respect to nourishment, as in the preceding experiments; it was also killed at half-past ten o'clock.—Crop filled with grain, humid, and very much swollen, appearing softened. The gizzard containing many fragments of vetches broken down, and much more altered than in the above-named experiments.

